# The Legislature and the Profession

**Published:** 1899-04

**Authors:** 


### THE LEGISLATURE AND THE PROFESSION.

THE present session of the legislature is doing more than its usual share of bill-introducing and reporting-from-committee, of measures, both favorable and unfavorable to the medical profession and a close scrutiny of all bills and a determined opposition to the vicious ones is necessary by the legislative committee of the various state medical societies. Some of the bills are of decided merit and the JOURNAL hopes them to become enacted into laws; as such may be mentioned the bill of Senator Davis making an appropriation for a state consumption hospital in the Adirondacks, which has been thoroughly agitated in these columns and its humanitarian features are self-evident.

The bill making further appropriation for the continuance of the state cancer laboratory in Buffalo seems destined to pass and the work begun will not suffer any from lack of state support. Some of the new bills introduced and which are meeting great opposition are the Collier bill, repealing section 1226 of the charter of the City of New York, having for its object the stopping of the sales of surplus lymph and antitoxins by the board of health of New York City. This bill allows the board of health to continue the manufacture of antitoxin and its free distribution to the indigent sick, but prevents it from conducting a commercial industry in competition with the trade.

Another measure which is in the line of reform and good usage is Mr. Ten Eyck's bill, making it compulsory for the manufacturers of any patent, proprietary or other medicine putting up prescriptions to label the vial, box, parcel or package with a list of the ingredients entering into the composition. The bill has been reported favorably by the public health committee of the assembly.

The opposition to such a measure is so strong and well organised that its final passage, much as it is desired, yet seems exceedingly doubtful. The powerful opposition made by these manufacturers before congress at the time of the passage of the war-revenue bill compelling a decided reduction in the stamp tax on these goods, will no doubt be brought to bear upon the legislature. Several bills have been introduced, especially by Mr. Costello in the assembly and by Mr. Sullivan in the senate, forbidding the manufacture of clothing in tenement houses, unless licensed by the state factory inspector and another requiring all such garments to be labeled "tenement-house made," both having in view the sweat-shop evil and the liable infection of such garments with the bacilli of tuberculosis. Both measures are excellent ones and are in a fair way to become laws. Some of the obnoxious or rather ill-advised measures are several looking toward a restriction of power of the state board of charities over the institutes for the deaf mutes and the blind and over the Gerry society in New York City. The important work accomplished by this board, its freedom from bias and partiality should cause institutions and societies to welcome its visitation and supervision instead of repelling them by act of legislature.

Of course, there can be no legislature without some members who must do something for their constitutents, even if their measures never leave the committee rooms. Such measures never see the light of day; neither do they deserve it. For instance, the bill requiring every theatre to have a physician present at every performance might be necessary at times, depending of course upon the performance and especially this winter at one of our best theatres, but as a rule, Buffalo audiences, with the exception of some mental nausea, have been quite free from the physician's care while in the play-house.

The introduction of so many absurd and grotesque measures only retards the progress of legislative work and the suggestion made at Albany this winter that all bills to be acted upon must first be submitted to a committee before introduction is a wise and timely one.

